# Enhancing *Deinagkistrodon acutus* antivenom potency through acutolysin A-targeted antibody supplementation

**DOI:** 10.1371/journal.pntd.0013847

**Published:** 2025-12-12

**Authors:** Chien-Chun Liu, Yu-Shao Chou, Yi-Xiu Yang, Yung-Chin Hsiao, Lichieh Julie Chu, Chi Yang, Zhongyi Yan, Renchao Peng, Hsiao-Yun Chao, Min-Xian Li, Cho-Ju Wu

**Affiliations:** 1 The School of Public Health and Medical Technology, Xiamen Medical College, Xiamen, Fujian, China; 2 Engineering Research Center of Natural Cosmeceuticals College of Fujian Province, Xiamen Medical College, Xiamen, Fujian, China; 3 Molecular Medicine Research Center, Chang Gung University, Taoyuan, Taiwan; 4 Department of Emergency Medicine, En Chu Kong Hospital, New Taipei City, Taiwan; 5 College of Medicine, Chang Gung University, Taoyuan, Taiwan; 6 Graduate Institute of Biomedical Sciences, College of Medicine, Chang Gung University, Taoyuan, Taiwan; 7 Department of Surgery, Chang Gung Memorial Hospital at Linkou, Taoyuan, Taiwan; 8 Department of Emergency Medicine, Chang Gung Memorial Hospital at Linkou, Taoyuan, Taiwan; 9 Department of Hepatobiliary Surgery, Xiamen Chang Gung Hospital Hua Qiao University, Xiamen, Fujian, China; Fundação de Medicina Tropical Doutor Heitor Vieira Dourado: Fundacao de Medicina Tropical Doutor Heitor Vieira Dourado, BRAZIL

## Abstract

**Background:**

Antivenom remains the standard and most effective treatment for snakebite envenomation, yet batch-to-batch variability in neutralization potency, even under standardized immunization protocols, continues to present a significant challenge in antivenom production worldwide. This issue is particularly evident in the manufacturing of *Deinagkistrodon acutus* antivenom in Taiwan, where some hyperimmunized equines fail to achieve the required potency despite identical conditions. Previous studies identified a lower antibody titer against acutolysin A—a key metalloproteinase toxin in *D. acutus* venom—as a potential contributor to reduced efficacy.

**Methodology/principal findings:**

In this study, acutolysin A was purified from *D. acutus* venom using a two-step HPLC workflow, and specific antibodies were isolated from high-potency equine plasma via affinity chromatography. When supplemented into low-potency plasma, these antibodies markedly improved survival in a murine lethality model, providing direct in vivo evidence that anti-acutolysin A antibodies are a critical determinant of antivenom efficacy. To explore strategies for enhancing these antibody levels, we evaluated a supplemental boosting approach in which purified acutolysin A replaced crude venom during later immunization rounds. This regimen significantly increased titers against acutolysin A but reduced immunoreactivity toward other venom proteins, raising concerns about narrowing antigenic breadth.

**Conclusions/significance:**

Collectively, our results highlight anti-acutolysin A antibodies as key determinants of *D. acutus* antivenom potency and demonstrate that their targeted supplementation can enhance neutralization efficacy, supporting antibody-based enhancement as a potential strategy to improve the potency of *D. acutus* antivenoms.

## Author summary

Snakebite is a serious health problem in many parts of the world, and antivenom is the only proven treatment. However, the strength of antivenom can vary between production batches, even when prepared in the same way. This inconsistency has been a challenge in Taiwan, where antivenom is made against the venom of the hundred-pace viper (*Deinagkistrodon acutus*). We investigated a venom protein called acutolysin A, which causes severe tissue damage. Our results provide direct evidence that antibodies targeting acutolysin A are a key factor in whether antivenom works effectively. By isolating these antibodies and adding them to weaker antivenom, we restored protection in mice, showing their central role in reducing venom toxicity. We also tested, using a mouse model, whether boosting with purified acutolysin A during immunization could raise antibody levels. This strategy successfully increased antibodies against acutolysin A but lowered responses to other venom proteins, indicating that a careful balance is needed between specificity and broad coverage. Together, these findings suggest that supplementing antivenoms with selected antibodies could be a simple way to improve their reliability and effectiveness for snakebite victims.

## Introduction

Snakebite envenomation is a globally recognized medical concern and is classified by the World Health Organization as a neglected tropical disease due to its significant morbidity and mortality burden [1]. Currently, the mainstay of treatment is antivenom therapy, which consists of antibodies derived from the serum of immunized animals such as horses or sheep [2]. These antibodies neutralize toxic venom components and mitigate the onset of systemic and local symptoms following envenomation.

To produce antivenom, animals are repeatedly immunized with snake venom to elicit a robust antibody response [3]. Once antibody titers reach a neutralization threshold, large volumes of plasma are harvested, from which immunoglobulins are purified for therapeutic use [4]. Despite decades of advancement since the concept of antivenom was first introduced by Henry Sewall in 1887 [5], the development of highly potent antivenoms remains challenging due to antigenic competition among different venom components. Even when subjected to identical immunization protocols, some hyperimmunized animals fail to achieve adequate neutralization capacity [6]. These unqualified animals must be excluded, leading to financial loss and inefficiencies in production due to the time-intensive screening process required to identify high-potency plasma donors.

A critical factor contributing to this limitation is the use of whole venom as the immunogen. While comprehensive in antigen content, whole venom may contain highly toxic components with poor immunogenicity. Consequently, antibodies produced may disproportionately target non-toxic proteins, thereby reducing overall neutralization efficacy [7]. Moreover, immune responses skewed toward irrelevant antigens may dilute the production of neutralizing antibodies against key toxins [8]. For example, in *Naja atra* (Taiwanese cobra) envenomation, cytotoxins (CTXs)—the major components responsible for local necrosis—exhibit relatively low immunogenicity despite their pathological importance [9]. As a result, CTX-specific antibody titers in hyperimmunized horses are often insufficient for effective neutralization [10,11]. These findings suggest that whole venom-based immunization may obscure immune responses to dominant toxins, ultimately limiting the potency of the final antivenom.

In Taiwan, the production of snake antivenom is solely managed by the Centers for Disease Control (CDC), which relies on equines for antibody generation [12]. The limitations of whole venom immunization are particularly evident in the production of *Deinagkistrodon acutus* antivenom. Despite repeated immunization cycles, a substantial proportion of equine plasma samples fail to meet the neutralization standards. Prior immunoprofiling studies have identified acutolysin A, the most hemorrhagic component in *D. acutus* venom [13], as a major differentiating antigen between high-potency plasma (> 60 Tanaka units/mL) and low-potency plasma (< 60 Tanaka units/mL) [14], with the high-potency group showing significantly greater neutralization activity. These data suggest that insufficient anti-acutolysin A antibody levels may be a principal determinant of suboptimal antivenom efficacy. However, this hypothesis remains to be confirmed through in vivo validation.

To overcome this limitation, several strategies have been proposed, including the supplementation of traditional antivenom with targeted anti-toxin agents such as monoclonal antibodies or aptamers [15–18], as well as the incorporation of additional immunization boosts with purified toxin components like acutolysin A during the immunization process [19]. While conceptually promising, these approaches require further experimental validation before practical implementation.

In this study, we hypothesized that antibodies targeting acutolysin A play a critical role in the neutralization potency of *D. acutus* antivenom. To test this hypothesis, we first purified acutolysin A from crude venom and isolated anti-acutolysin A antibodies from high-potency equine plasma to directly assess their contribution to venom neutralization in a murine model. We further evaluated whether boosting with purified acutolysin A during immunization could enhance antibody titers against this key toxin. By combining these approaches, we aimed to clarify the functional importance of anti-acutolysin A antibodies and to explore practical strategies for improving antivenom potency and consistency. This work provides mechanistic insight that may guide future optimization of antivenom production and targeted immunization protocols.

## Materials and methods

### Ethics statement

Experiments involving the care and injection of mice with various venoms were reviewed and approved by the Institutional Animal Care and Use Committee of Chang Gung University (Permit Number: CGU112-101). The protocol of animal study on mice was based on the guidelines given by the law of animal protection act in Taiwan and the Council for International Organizations of Medical Sciences (CIOMS) [20].

### Snake venom and hyperimmunized horse plasma

Crude *D. acutus* venom and hyperimmunized equine plasma samples, including both high-potency (> 60 Tanaka units/mL) and low-potency (< 60 Tanaka units/mL) groups, were provided by the Centers for Disease Control, Taipei, Taiwan. Venom was lyophilized and stored at −20℃. Horse plasma was stored at -80℃ before use. Neutralization potency was expressed in Tanaka units, a standard measure used in Taiwan for evaluating antivenom efficacy. One Tanaka unit corresponds to the volume of antivenom required to fully neutralize one minimum lethal dose (MLD) of *D. acutus* venom in a mouse model. Potency values (Tanaka units/mL) were determined as previously described [14].

### Isolation of acutolysin A from *D. acutus* venom

A two-dimensional liquid chromatography procedure was employed for the purification of acutolysin A from *D. acutus* venom. Briefly, 100 mg of crude venom was dissolved in a 10 mL of 0.02 M Tris-HCl buffer (pH 8.0), and loaded onto a DEAE Sepharose Fast Flow column (Cytiva, Marlborough, MA, USA) pre-equilibrated with the same buffer. Chromatographic separation was achieved using a continuous linear gradient of solvents A and B (0.02 M Tris containing 0.5 M NaCl) with the following gradient profile: 0–15% solution B over 10 min, followed by 15–35% solvent B over 40 min, and 35–100% solvent B over 5 min. The flow rate was maintained at 2 mL/min, and elution fractions were collected at 1 mL intervals. Protein peaks were detected by monitoring absorbance at 280 nm. Subsequently, the fraction containing acutolysin A was collected, concentrated, and applied to a Superdex 75 increase 10/300 GL column (Cytiva, Marlborough, Massachusetts, USA). The column was equilibrated and eluted with a 0.15 M NaCl solution. The flow rate was set to 0.4 mL/min, with elution fractions collected at 0.4 mL intervals. Protein peaks were again detected by monitoring absorbance at 280 nm.

### Affinity purification of anti-acutolysin A antibodies

The methods used for affinity purification of antibodies were described previously [21]. Briefly, the *D. acutus* venom-hyperimmunized equine plasma (Tanaka unit > 60) was cycled through the acutolysin A-coupled Sepharose 4B column (1 cm × 5 cm). The bound antibodies were eluted from the column with 20 mL of 100 mM triethylamine (pH 11.5), and the eluted fractions (1 ml per tube) were collected in tubes containing 0.1 mL of 1 M Tris/HCl (pH 8.0). The purified anti-acutolysin A antibodies were concentrated to approximately 1 mL, dialyzed against PBS containing 50% (v/v) glycerol and 0.04% NaN_3_, and then stored at −20 °C.

### Animals

Experiments were performed on three-week-old littermate ICR (CD1) mice with a defined weight range (15–18 g). Mice were maintained in specific pathogen-free conditions. They were housed in a 12:12 h light dark cycle at a temperature of 22℃ and a humidity level of 60 – 70%. Animals had ad libitum access to food and water.

### Evaluation of the neutralization potency in rodent models

The analysis method applied to determine neutralization potency at Taiwanese CDC was followed and modified [22,23]. Briefly, the minimal lethal dose (MLD) of *D. acutus* venom was determined as 60.8 μg, which displayed the lowest dose of venom-inducing lethality in all injected mice. One-half milliliter of PBS containing 4 MLD of *D. acutus* venom was mixed with 0.5 mL of 3-fold dilution horse plasma and incubated at 37℃ for 1 h. Then, 0.2 mL of mixture was intraperitoneally administrated into group of ICR mice (n = 3). The survival rate was recorded at 48 h post-injection.

### Immunization of mice with snake venom proteins

**Fig 1:**
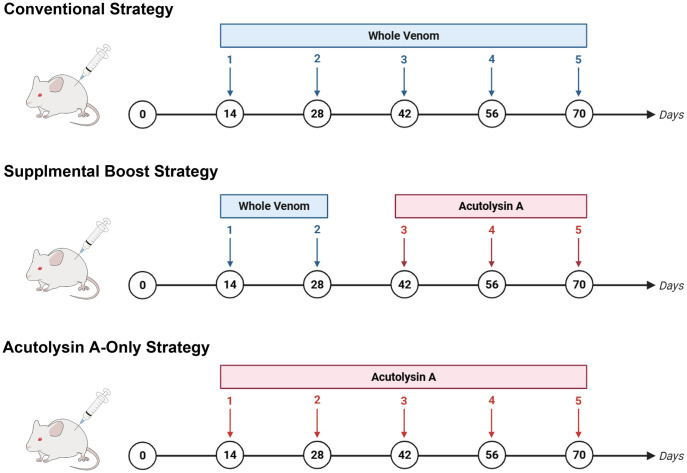
Immunization workflows and timelines for conventional, supplemental boosting, and acutolysin A–only strategies. Schematic overview of the immunization schedules used in this study. Mice were immunized according to one of three regimens: conventional whole venom immunization, supplemental boosting with acutolysin A after two whole venom immunizations (Supplemental Boost), or acutolysin A–only immunization.

*D. acutus* whole venom or purified acutolysin A was used as the antigen to raise antibodies in groups of ICR mice (n = 3 per group). Venom protein (10 ng) in 100 µL PBS was heated at 95 °C for 5 minutes and then mixed with an equal volume of Freund’s adjuvant. The antigen–adjuvant mixture was administered by intraperitoneal injection. The immunization schedule of each group is shown in Fig 1. Plasma samples were collected from the facial vein 10 days after immunization and stored at −80 °C until use.

### Indirect-enzyme-linked immunosorbent assay (Indirect ELISA)

The method was performed with modifications based on a previously described [14], and the appropriate serum dilution factor was determined using a serum titration assay (**S1 Fig**). The *D. acutus* venom or acutolysin A (10 ng) was diluted in 50 µL PBS and coated onto 96-well polystyrene microplates (Corning Inc., Corning, New York, USA) by incubating at 4℃ overnight. The plates were washed six times with 100 µL of PBST (contain 0.1% Tween-20) and blocked with 100 µL of 1% ovalbumin in PBS at room temperature for 2 h. After repeating the washing step for six times, murine plasma was diluted (1:5,000) in PBS and added to each well, respectively. After incubation at room temperature for 2 h, the excess antibodies were removed by washing 6 times with PBST. Then, goat anti-mouse IgG conjugated with horseradish peroxidase (HRP) (Bethyl Laboratories, Montgomery, TX, USA) was added to each well and incubated at room temperature for 1 h. After finally washing each well 6 times with PBST, 50 µL of tetramethylbenzidine (TMB) substrate (Clinical Science Products Inc., Mansﬁeld, MA, USA) was reacted with each well for 10 minutes. The reaction was terminated with 25 µL of 2N H_2_SO_4_ (J.T Baker, Radnor, PA, USA) and absorbance of each well measured with a SpectraMax M5 microplate reader (Molecular Devices, San Jose, CA, USA) at excitation and emission wavelengths of 450 and 540 nm, respectively. Each assay condition was performed in triplicate.

### Sodium dodecyl sulfate-polyacrylamide gel electrophoresis (SDS-PAGE)

The protein concentration of each sample was measured using the Pierce BCA Protein Assay Kit (Thermo Fisher Scientific, Waltham, Massachusetts, USA). Ten microgram of whole venom, or two microgram of fractionated protein, was analyzed by SDS-PAGE under reducing condition. Briefly, samples were dissolved in sample buffer (125 mM Tris, 25% glycerol, 10% 2-mercaptoethanol, 4% SDS, 0.05% bromophenol blue) and heated at 95℃ for 5 minutes. Samples were then loaded onto a 15% gel and further separated by SDS-PAGE. The location of proteins in SDS-PAGE gels was visualized by Coomassie Brilliant Blue staining.

### Western blot analysis

The venom protein was resolved by SDS-PAGE gel, transferred onto polyvinylidene difluoride (PVDF) membranes, and then probed with 1:500 (v/v) dilution of equine plasma at 4℃ for 16 h. Antivenom-reactive proteins were detected by incubating with rabbit anti-horse IgG conjugated with HRP for 1 h, and visualized using the ECL system.

### Statistical analysis

Statistical analysis was performed using Log-rank test and one-way ANOVA followed by Tukey’s Multiple comparison test. Both analyses were performed using GraphPad Prism 9 software (La Jolla, California, USA). Differences were considered statistically significant at *p*-values < 0.05.

## Results

### Purification of acutolysin A from *D. acutus* venom via two-dimensional chromatography

**Fig 2:**
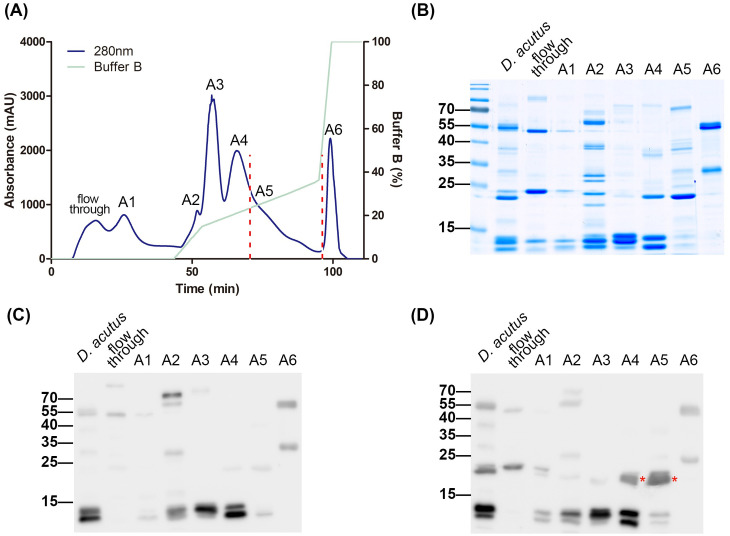
Fractionation of *D. acutus* venom by DEAE-HPLC. **(A)** Crude venom was fractionated by DEAE-HPLC, and elution was monitored at 280 nm. **(B)** Six protein fractions (A1–A6) were analyzed by SDS-PAGE and visualized with Coomassie blue staining. **(C, D)** Western blotting of fractions A1–A5 was performed using low-potency **(C)** and high-potency **(D)** equine plasma. The red star indicates the putative location of acutolysin **A.**

To obtain highly purified acutolysin A, *D. acutus* venom was fractionated using a two-step chromatography workflow. In the first dimension, crude venom was separated by DEAE-HPLC under a discontinuous three-step gradient, and protein elution was monitored at 280 nm. This process resolved the venom into six distinct fractions, designated A1–A6 (**Fig 2A**). SDS-PAGE analysis of these fractions, followed by Coomassie blue staining, revealed prominent protein bands in multiple fractions (**Fig 2B**). Given that acutolysin A has a reported molecular weight of ~25 kDa, fractions A4 and A5 were of particular interest, each containing a distinct 15–25 kDa band.

To assess the identity of these bands, western blotting was performed using plasma from two hyperimmunized horses with contrasting neutralization potencies: high potency (> 60 Tanaka units/mL) and low potency (< 60 Tanaka units/mL). Immunoblot analysis showed that protein bands within the 15–25 kDa range in fractions A4 and A5 were specifically recognized by high-potency plasma but not by low-potency plasma (**Fig 2C and 2D**). This selective recognition strongly suggested that acutolysin A was concentrated within these fractions.

**Fig 3:**
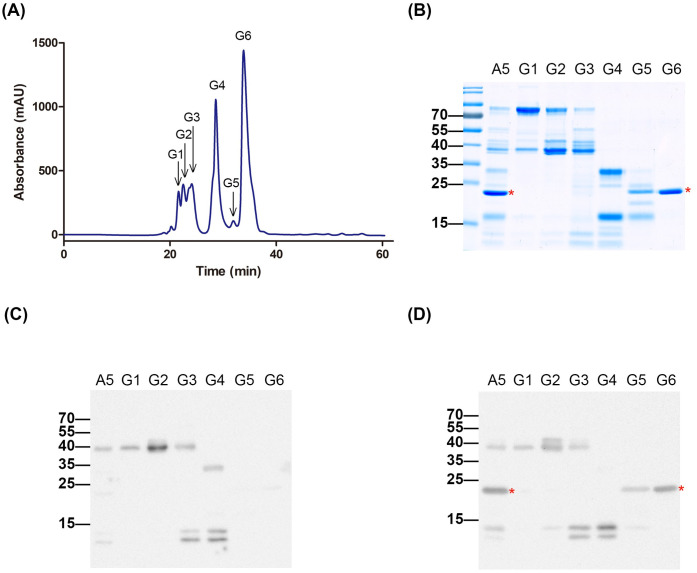
Purification of acutolysin A by gel filtration chromatography. **(A)** Fraction A5 from Fig 1 was further separated by gel filtration chromatography, with elution monitored at 280 nm. **(B)** Six subfractions (G1–G6) were analyzed by SDS-PAGE and stained with Coomassie blue. **(C, D)** Western blotting of fraction A5 and subfractions G1–G6 using low-potency **(C)** and high-potency **(D)** plasma. The red star indicates the acutolysin A protein band.

In the second purification step, fraction A5 was subjected to gel filtration chromatography, producing six subfractions (G1–G6) (**Fig 3A**). SDS-PAGE analysis indicated that fraction G6 contained a dominant protein band within the 15–25 kDa range (**Fig 3B**). Immunoblotting confirmed that this band was recognized exclusively by high-potency plasma and not by low-potency plasma (**Fig 3C and 3D**), mirroring the pattern observed in the first dimension. The purity of acutolysin A in fraction G6 exceeded 95%, and LC-MS/MS analysis confirmed its identity. Overall, approximately 2 mg of purified acutolysin A was obtained from 100 mg of crude venom using this two-dimensional chromatography protocol. These results established fraction G6 as a highly purified acutolysin A preparation suitable for downstream antibody isolation and functional studies.

### Contribution of anti-acutolysin A antibodies to neutralization potency

**Fig 4:**
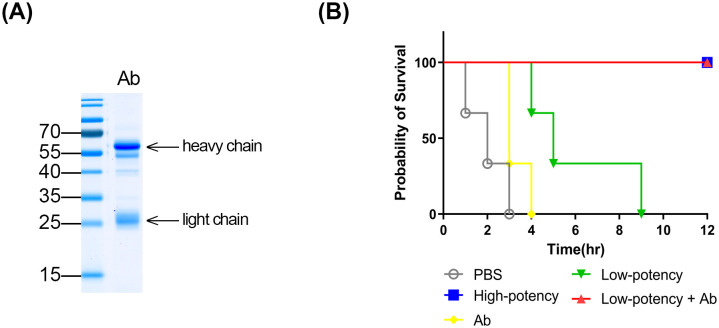
Contribution of anti-acutolysin A antibodies to neutralization potency. **(A)** SDS-PAGE profile of purified anti-acutolysin A antibodies, visualized with Coomassie blue staining. **(B)** Kaplan–Meier survival curves of mice (n = 3 per group) challenged with *D. acutus* venom and treated with PBS, high-potency plasma (High-potency), anti-acutolysin A antibody (Ab), low-potency plasma (Low-potency), or low-potency plasma with supplemental anti-acutolysin A antibodies (Low-potency + Ab), respectively. Survival was monitored hourly for 12 **h.** Statistical comparison between groups was performed using the log-rank test.

The selective recognition of acutolysin A by high-potency plasma observed in the preceding experiments (**Fig 3**) suggested that antibodies targeting this metalloproteinase may be critical determinants of *D. acutus* antivenom efficacy. To test this hypothesis directly in vivo, anti-acutolysin A antibodies were purified from high-potency equine plasma via affinity chromatography using an acutolysin A–coupled Sepharose column, yielding approximately 1 mg of antibody per milliliter of plasma (**S1 Table**). SDS-PAGE analysis of the purified fraction revealed clear IgG heavy chains (55–70 kDa) and light chains (~25 kDa), confirming high antibody purity (**Fig 4A**).

To assess functional contribution, these antibodies were supplemented into low-potency plasma, and the mixtures were tested in a murine lethality model. In untreated mice, *D. acutus* venom caused 100% mortality within 3 h. Administration of high-potency plasma completely protected all mice, whereas low-potency plasma only prolonged survival to 4–9 h. Notably, supplementation of low-potency plasma with purified anti-acutolysin A antibodies restored protection to 100% survival at 12 h (**Fig 4B**).

These findings provide direct in vivo evidence that the presence and concentration of anti-acutolysin A antibodies are critical determinants of antivenom efficacy against *D. acutus* venom. Furthermore, they suggest that targeted supplementation of specific antibodies could rescue suboptimal plasma batches, potentially improving production efficiency by reducing the need to discard low-potency plasma.

### Enhancing anti-acutolysin A antibody titers

**Fig 5:**
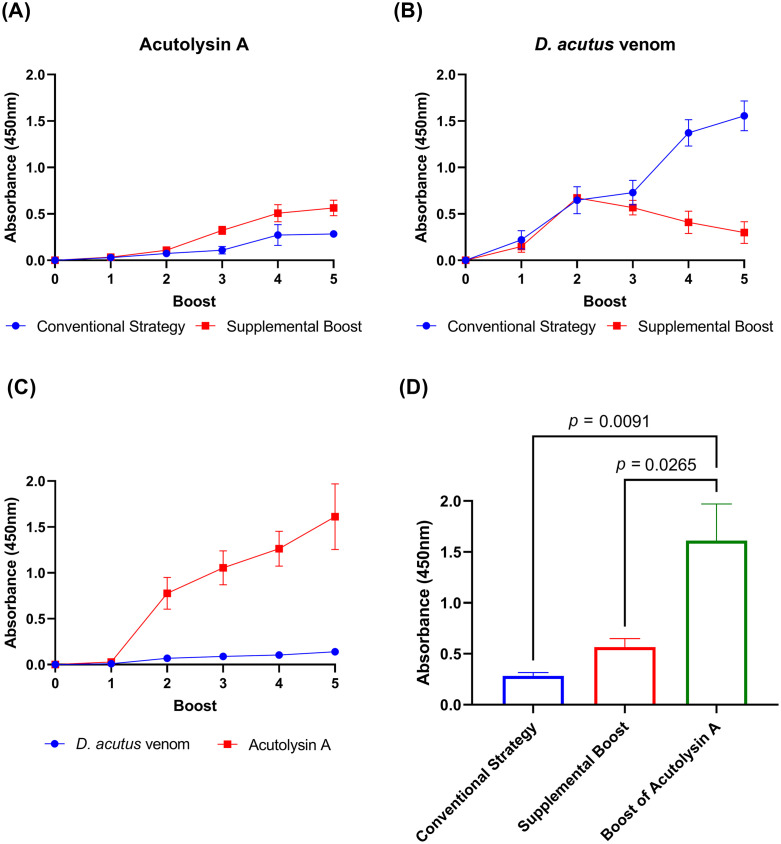
Immune responses to *D. acutus* venom and acutolysin A under different boosting strategies. **(A, B)** Indirect ELISA was used to assess plasma affinity for (A) acutolysin A and **(B)** *D. acutus* venom. **(C)** In a separate regimen, mice were boosted exclusively with acutolysin A for five consecutive rounds, and plasma from each round was analyzed for reactivity against both acutolysin A and *D. acutus* venom. **(D)** Comparison of anti-acutolysin A titers in plasma collected after the fifth round from all three immunization strategies. Data points and bars represent mean ± SD of triplicate measurements. Statistical significance was assessed using one-way ANOVA.

Given the central role of anti-acutolysin A antibodies in neutralization potency, we evaluated strategies aimed at increasing their titers. In the supplemental boosting approach, mice were first immunized twice with whole *D. acutus* venom, then administered a third immunization containing purified acutolysin A instead of crude venom (**Fig 1**). This strategy resulted in a twofold increase in anti-acutolysin A titers compared with the conventional whole venom protocol (**Fig 5A**). These data indicate that incorporating a targeted antigenic boost can selectively enhance antibody production against a key toxin. However, indirect ELISA using whole *D. acutus* venom as the coating antigen revealed a significant reduction in antibody reactivity toward other venom components in the supplemental boost group (**Fig 5B**), a pattern that was similarly observed in the western blotting results (**S2A Fig**). This finding suggests a narrowing of antigenic breadth, which could potentially compromise overall neutralization if other toxic components contribute to lethality.

To further investigate the effect of antigen specificity on titer magnitude, a separate cohort of mice was immunized exclusively with purified acutolysin A over five consecutive rounds (**Fig 1**). In this regimen, anti-acutolysin A titers increased markedly after the second round and continued to rise steadily, reaching 1.5-fold above the second-round level by the fifth round (**Fig 5C**). This continuous elevation suggests that repeated exposure to the same purified antigen maintains a focused and robust humoral response.

When antibody titers against acutolysin A were compared after five rounds of immunization across the three strategies (conventional whole venom, supplemental boosting, and acutolysin A–only), the acutolysin A–only group achieved titers approximately threefold higher than those of the supplemental boost group and substantially higher than those of the conventional group (**Figs 5D and** S2B). These differences were statistically significant, with the acutolysin A–only group showing higher titers than the conventional group (*p* = 0.0091) and the supplemental boost group (*p* = 0.0265). These findings confirm that exclusive immunization with acutolysin A produces the most potent and sustained antibody response against this specific antigen.

Nevertheless, while acutolysin A–only immunization maximized antigen-specific titers, it is important to note that such a focused response may come at the expense of cross-reactivity to other toxic components. This trade-off, also observed with the supplemental boost strategy, highlights the need to balance specificity and breadth when designing immunization regimens for antivenom production.

## Discussion

Variation in the potency of snake antivenom produced by different equines under identical immunization conditions remains a major challenge in venom therapy worldwide [24,25]. This issue is also encountered in the production of *D. acutus* antivenom in Taiwan. Previous work comparing the immune recognition profiles of high-potency and low-potency equine plasma identified the antibody titer against acutolysin A as a potential determinant of neutralization efficacy [14]. However, the contribution of anti-acutolysin A antibodies to in vivo protection had not been experimentally verified.

In the present study, we isolated acutolysin A from *D. acutus* venom using two-step HPLC, followed by purification of anti-acutolysin A antibodies from hyperimmunized equine plasma through affinity chromatography. Supplementation of these purified antibodies into low-potency plasma significantly enhanced its neutralization potency in a murine lethality model. These results provide direct in vivo evidence that the abundance of anti-acutolysin A antibodies in equine plasma plays a critical role in preventing *D. acutus*-induced lethality. This finding may contribute to improving the efficiency of antivenom production and support future investigations into the clinical management of *D. acutus* envenoming.

Several innovative approaches have been proposed to address the broader challenge of insufficient titers of antibodies against major toxin proteins in antivenom preparations [26]. Two principal strategies include [1] administering booster injections of recombinant or purified toxin components during the hyperimmunization process, and [2] incorporating anti-toxin biomolecules into conventional antisera to create recombinant antivenoms.

The booster injection approach follows conventional immunization protocols but substitutes crude venom with purified or recombinant forms of major toxins. This targeted strategy can elicit a more focused immune response, leading to increased production of therapeutically relevant antibodies with higher specificity and potency [27–29]. In addition, the use of recombinant or native toxin proteins ensures more consistent immunogen quality, potentially improving the reliability of antibody yields. This method is straightforward to implement in existing production systems and may provide an effective solution to insufficient antivenom potency against key toxins.

Another promising approach is the development of recombinant antivenoms by supplementing conventional antisera with additional anti-toxin agents, such as monoclonal antibodies, aptamers, or small-molecule inhibitors. These additives can significantly boost the neutralization potency of otherwise suboptimal antivenoms [30–33], yielding preparations with enhanced specificity toward major toxins while preserving the overall serum composition [34].

In our study, we evaluated an additional boost strategy targeting acutolysin A during immunization. Mice that received an acutolysin A boost following hyperimmunization with *D. acutus* venom developed significantly elevated titers of anti-acutolysin A antibodies, mimicking the immune enhancement expected in production horses. However, this strategy was accompanied by a notable reduction in antibody titers against other venom proteins, raising concerns about narrowing antigenic breadth. The potential impact of this shift on overall neutralization potency remains uncertain, especially given that the contribution of other *D. acutus* venom components to lethality has yet to be fully defined. Further work is needed to determine whether the increased specificity toward acutolysin A offsets any loss in coverage against other toxins.

An alternative strategy supported by our findings is the direct incorporation of anti-acutolysin A antibodies into conventional antiserum to create a recombinant antivenom. This approach significantly improved neutralization potency in vivo without altering the antibody composition of the base serum, thereby preserving broad-spectrum activity. Maintaining this breadth of activity is crucial, as the complexity of venom composition and the interplay of multiple toxins in pathogenesis require coverage beyond a single antigen.

Beyond antibodies, other molecular scaffolds such as aptamers and small-molecule inhibitors offer additional advantages in recombinant antivenom development. These agents are cost-effective, exhibit low immunogenicity, and can be engineered for high specificity toward individual toxins. Aptamers or small molecules targeting acutolysin A could be explored as adjuncts or stand-alone therapeutics, potentially yielding antivenoms that are both more effective and safer.

Collectively, the results of this study provide direct in vivo evidence that antibodies targeting acutolysin A are a pivotal determinant of the neutralization potency of *D. acutus* antivenom. By isolating and supplementing these antibodies into low-potency equine plasma, we demonstrated a marked improvement in survival outcomes in a murine lethality model, underscoring their central role in mitigating venom-induced toxicity. These findings further highlight recombinant supplementation with specific antibodies as a straightforward and practical strategy to enhance antivenom efficacy, potentially improving consistency across production batches. Beyond confirming the importance of anti-acutolysin A antibodies, our results also reveal the potential and limitations of targeted immunization strategies. While supplemental boosting with purified acutolysin A effectively increased antibody titers against this key metalloproteinase, it concurrently reduced immune recognition of other venom components, suggesting that antigenic specificity must be carefully balanced with broad-spectrum coverage to ensure comprehensive neutralization.

Future studies should extend these findings toward clinical applications by evaluating antibody-based enhancement strategies in therapeutic contexts. In particular, region-specific investigations considering geographical variations in *D. acutus* venom composition and antivenom response will be essential to optimize treatment efficacy and guide the development of standardized, high-potency antivenoms. In addition, several limitations of this study should also be acknowledged. Due to funding constraints, immunological responses were not assessed directly in equines, which are the primary production animals for antivenom manufacturing. Instead, murine models were used to evaluate the effects of acutolysin A–specific boosting, and future validation in horses will be important. Furthermore, our neutralization assessment used a preincubation model in which venom was mixed with plasma prior to injection. Although this approach is suitable for initial characterization, it does not fully reflect clinical treatment conditions. Rescue experiments, in which venom is administered first and antivenom is delivered later, should be included in future work to better represent real-world therapeutic scenarios. Addressing these limitations will enhance the translational relevance of antibody supplementation strategies and support their potential integration into antivenom development pipelines.

## Supporting information

S1 Table Summary of anti-acutolysin A antibody yield from the purification process. (XLSX)

S1 Fig Titration assays of hyperimmunized murine sera obtained from different boosting strategies. (TIF)

S2 Fig Western blot analysis of venom antigens recognized by antibodies from different immunization protocols. The serum from different immunization strategies was probed with (A) whole *D. acutus* venom and (B) purified acutolysin A, respectively.
(TIF)

S1 Data Excel sheet containing raw data of Figs. (XLSX)
