# Supplementary figures and images for: Enhancing Deinagkistrodon acutus antivenom potency through acutolysin A-targeted antibody supplementation
Source: PLoS Negl Trop Dis. 2025 Dec 12;19(12):e0013847. doi: 10.1371/journal.pntd.0013847 (PMC12714209; doi:10.1371/journal.pntd.0013847)

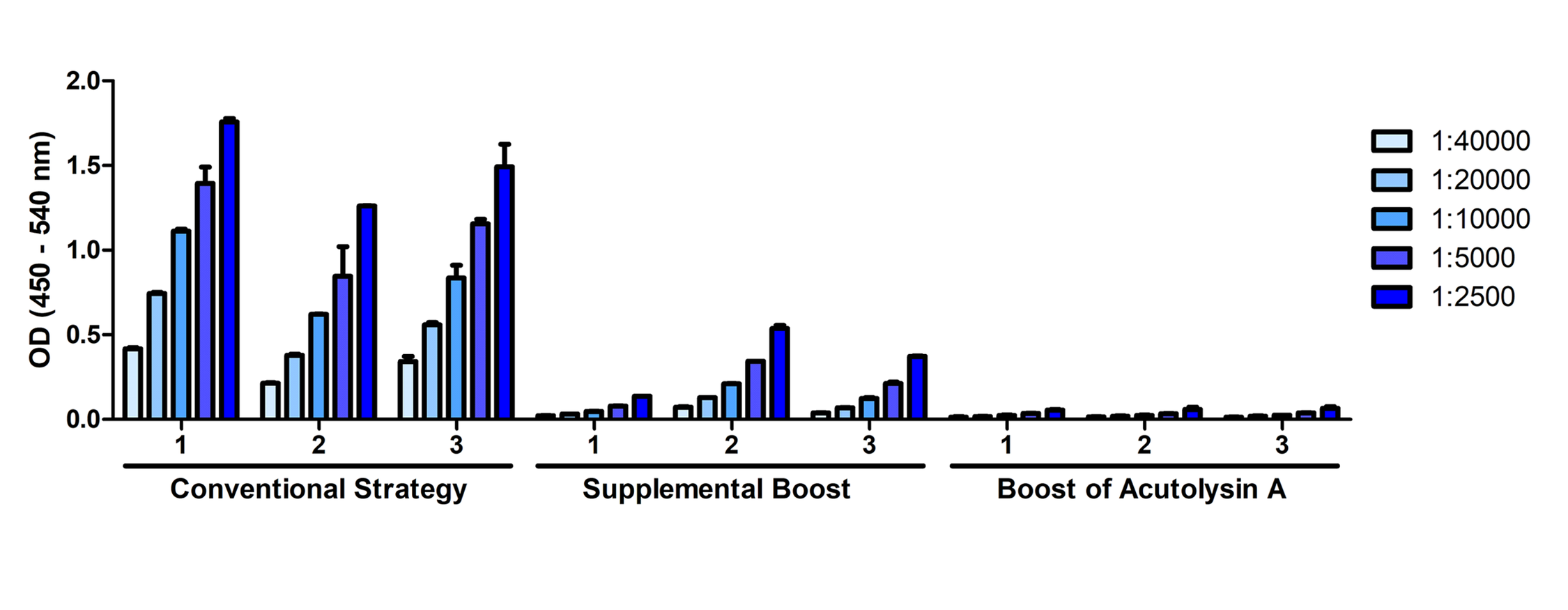

Supplement: S1 Fig — (TIF) [file pntd.0013847.s002.tif]

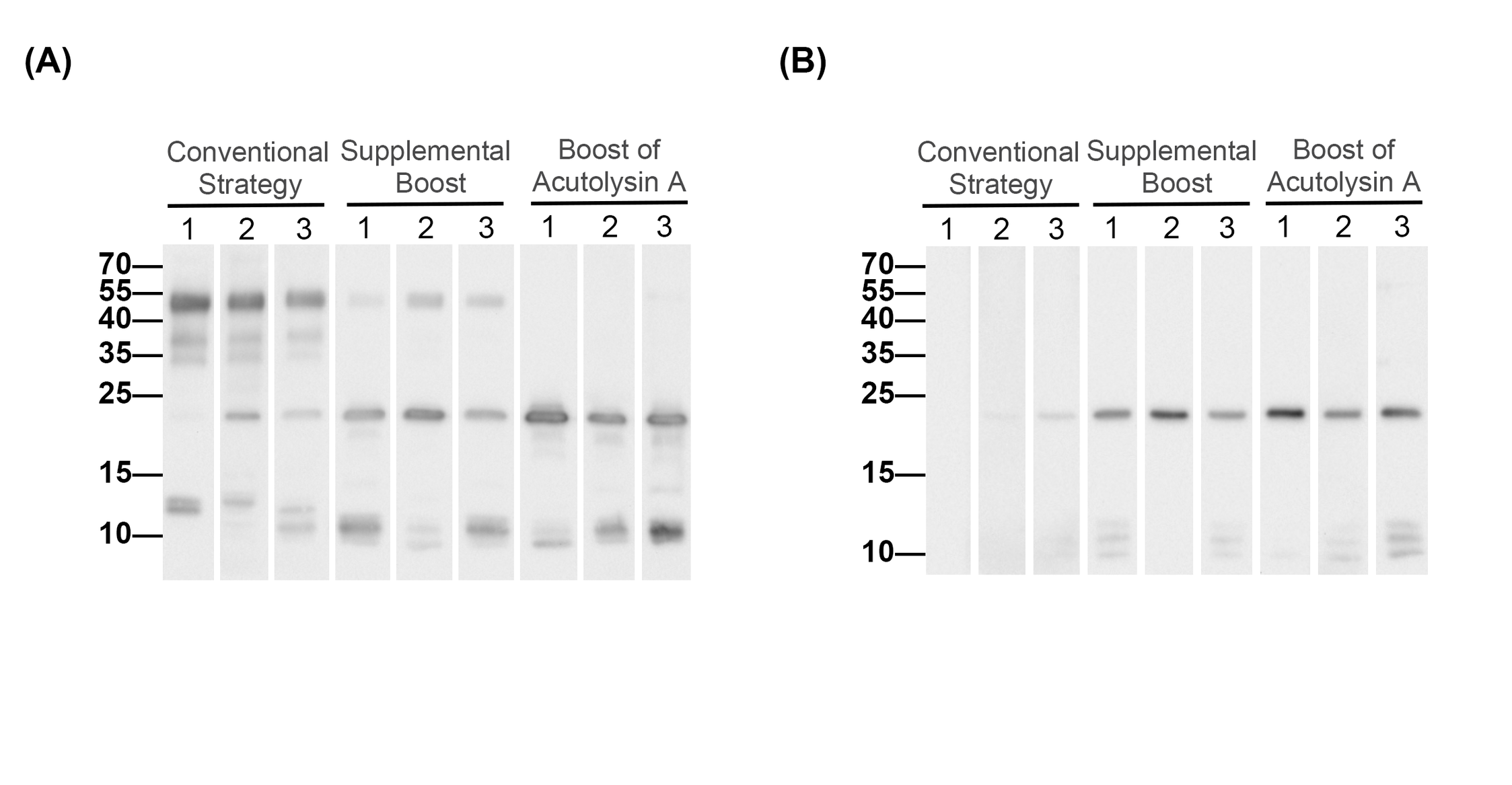

Supplement: S2 Fig — The serum from different immunization strategies was probed with (A) whole D. acutus venom and (B) purified acutolysin A, respectively. (TIF) [file pntd.0013847.s003.tif]
